# Play, art, music and exercise therapy impact on children with diabetes

**DOI:** 10.1007/s11845-021-02889-5

**Published:** 2022-01-17

**Authors:** Ioana Patricia Bacus, Husnain Mahomed, Anne-Marie Murphy, Muiriosa Connolly, Orla Neylon, Clodagh O’Gorman

**Affiliations:** 1 Department of Paediatrics, School of Medicine, University of Limerick, Limerick, Ireland; 2 Department of Paediatrics, University of Limerick, Limerick, Ireland

**Keywords:** Art therapy, Exercise therapy, Music therapy, Play therapy, Type 1 diabetes mellitus

## Abstract

Diabetes mellitus (DM) is a global public health issue. Type 1 diabetes (T1D) is the predominant diabetes type in children and always requires insulin therapy. The incidence rate of newly diagnosed T1D in children continues to increase in Ireland Roche et al. (Eur J Pediatr 175(12):1913-1919, 2016) and worldwide Patterson et al. (Diabetologia 62(3):408-417, 2019). The objective of this study was to conduct a literature review of the effects of various non-pharmacological therapeutic modalities on the control of diabetes in children. A literature review was performed using PubMed, Medline, Embase and Cochrane library to evaluate play, art, music and exercise therapy in the treatment of DM using the keywords: “paediatric”, “diabetes”, “play therapy”, “art therapy”, “music therapy” and “exercise therapy”. These search terms initially returned 270 cases, which resulted in a total of 11 papers being reviewed after eliminating duplicate or irrelevant papers. Literature review showed that all therapies have a positive impact on the child, but there is limited research looking at the impact of therapy on quantitative measures such as HbA1c or ‘time in range’.

## Introduction

The incidence of diabetes mellitus (DM) continues to rise worldwide and modelling suggests that this increase will continue, according to the World Health Organization (WHO) (https://www.who.int/) and Centers for Disease Control and Prevention (CDC) (https://www.cdc.gov/). Among youth in the USA, both type 1 diabetes (T1D) and type 2 Diabetes (T2D) are increasing rapidly. Between 2000 and 2009, the incidence of T1D among children increased by 21% (from 1.48 to 1.93 per 1000 children), while T2D increased by 30% (from 0.34 to 0.46 per 1000 children) during the same timeframe [3]. Furthermore, the associated health care costs for youth with DM are on average six times higher than for youth without DM [4]. While T1D is the most common in all children and young people, the incidence of T2D among teens is increasing significantly in teenagers [5]. Regardless of type, DM is a chronic condition with a requirement for lifelong management and can pose many challenges. Teenagers face unique challenges and developmental changes that can make self-management of DM difficult. They experience social, cognitive, and psychological pressures that can impact their chronic disease management. These challenges include peer pressure, fear of social stigma, risk-taking behaviours and the drive for independence from parents. Given the trend of reduced adherence and suboptimal glycaemic control that typically occurs during adolescence, clinicians and researchers must continue to find ways to effectively reach teenagers and encourage good diabetes management habits [6].

Play is normal behaviour for children, and play therapy specifically has been found to be an effective care strategy for chronically ill children. Play accomplishes many goals for children beyond simple pleasure, including the means to evolve and prepare themselves for adulthood. Play is an important part of the learning process, and psychologists and educationalists are quick to highlight the value of play in the development of children. When children play they explore their abilities and learn to adapt and work within their environment. Play is also a key part of children’s development regarding maturation of communication skills and understanding of their own bodies [7].

In art therapy there have recently been many qualitative studies that have focused on DM and its related issues [8, 9]. Although it is difficult to quantify the possible impacts on glycaemic control, the meaning that patients apply to an illness does affect how they rate their overall health [10]. Additionally, these perceptions may influence treatment effectiveness, psychological symptoms, coping and outcomes [10]. Arts are increasingly being utilized in health care and their use in psychotherapeutic contexts has been extensively studied previously, assisting patients to communicate their feelings more effectively. Camic discusses extensively how and why these approaches are useful from the view of health psychology, and Lane et al. discusses how these approaches have been actively used in nursing practice [11, 12]. Stuckey and Nobel reviewed literature that looked into the relationship between creative arts and health outcomes, specifically in music engagement, visual arts therapy and creative expression and writing [13]. In all of these areas, they discovered clear indicators that engagement in artistic activities has significant positive impacts.

Music therapy has been shown to be beneficial for the health of children in a variety of contexts ranging from neonatal care to autism spectrum disorder [14]. Music therapy has been used to help with non-verbal processing of information and emotions [14]. Additionally it has been shown to positively impact the patients’ mood and motivation leading to increased upper extremity function in neurorehabilitation [15]. Patients do not have to be musically gifted to be able to see an improvement in using music therapy [14].

Exercise therapy can affect endothelial function and in turn reduce the cardiovascular risk profile.

Regular physical activity has been shown to correct endothelial dysfunction in patients with chronic heart failure, hypercholesterolemia and polymetabolic syndrome [16]. A recent cross-sectional study indicated a strong correlation between the level of physical activity and vascular function in children with T1D [16].

## Methods

**Fig. 1:**
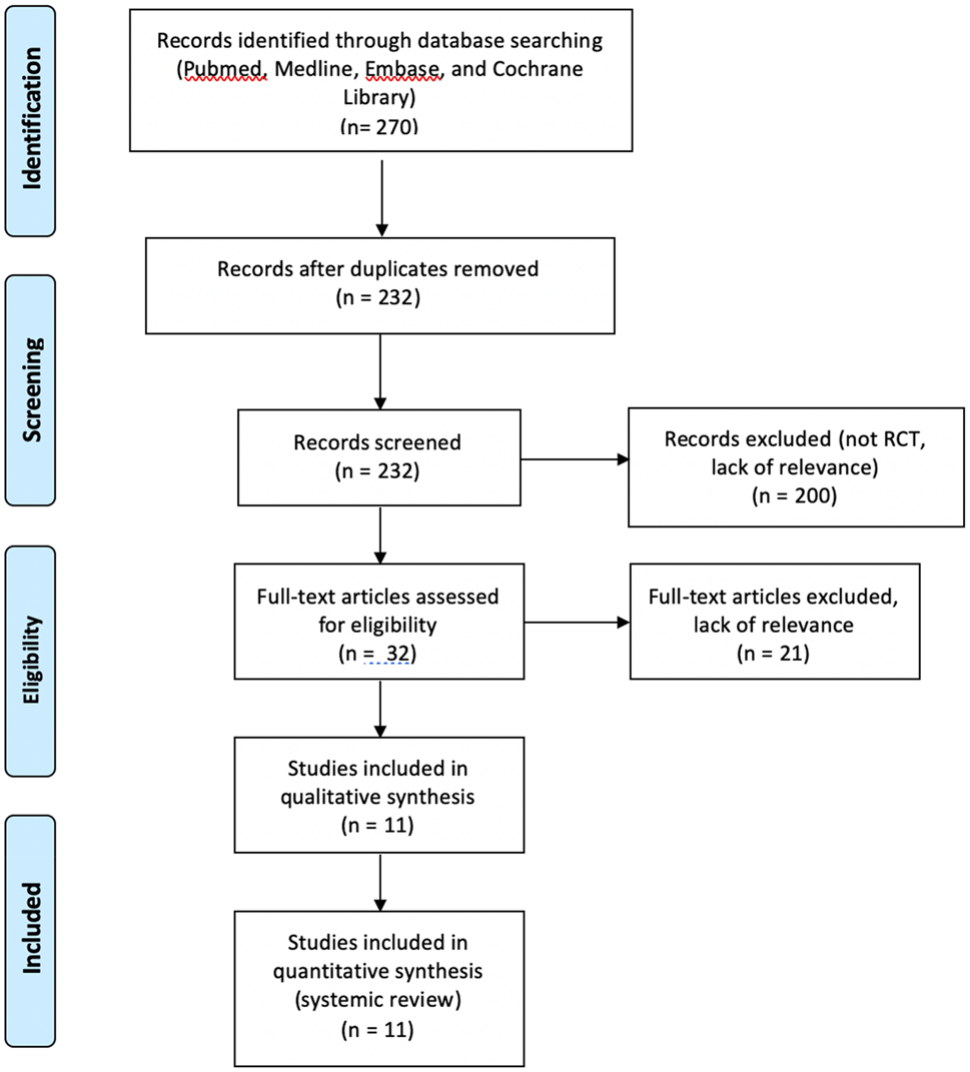
Methods of systematic review of literature. Using search terms “Play therapy”, “art therapy”, “music therapy”, “exercise therapy” and “diabetes” until January 2020. Limited to RCTs in paediatric population with T1DM

A literature search was conducted for randomized control trials using PubMed, Medline, Embase and Cochrane library. In particular, articles containing the medical subject headings (MeSH) type 1 diabetes mellitus, art therapy, play therapy, dance therapy and music therapy were retrieved. The inclusion criteria utilized incorporated studies published till January 2020. Using the search terms “play therapy”, “art therapy” “music therapy”, “exercise therapy” and “diabetes” limiting it to people under the age of 18, 270 papers were identified of which 38 were excluded for being duplicates and 11 were selected for inclusion (Fig. 1). Play therapy had the most published articles with 4 papers identified, followed by art therapy and music therapy with 3 papers each and music therapy with 1. Articles were selected on the basis that one of the four therapy modalities were used in children with diabetes to evaluate understanding of diabetes and/or improvement in glycaemic control. Papers were excluded if less than 50% of the participants were children. Feasibility studies were also excluded. Only English language papers were included.

## Results

### Play therapy (4 papers)

Henkemans et al. studied the use of technology in health education for children with T1D. Their study used a personal robot to quiz the children on educational questions relating to treatment of their diabetes. They found that the children were more responsive and engaged to education through the robot and perceived more enjoyment through the process. Although this level of enjoyment fell over time, it remained high compared to normal methods.

Pelicand et al. studied the impact of a therapeutic education program for children with diabetes. Over the course of 3 weeks during a summer camp, an educational program was implemented that was intended to both entertain the children while educating them on diabetes and treatment options to promote independence and health. The program was split into 7 workshops, lasting 1 h each. The results suggested that the children better understood the material compared to normal methods, and the program was perceived as good or very good by 13 out of the 14 children on a 5-point scale (very bad, bad, neutral, good, very good).

Pennafort et al. analysed the impact of using toys to help children with diabetes better understand glycaemic monitoring and insulin application. They had children perform these actions directly on dolls to help familiarize them with the procedures and improve their attitude towards them. They found that these actions helped to improve the ability of the children to independently perform these actions, becoming less afraid of needles and better understanding the need for insulin management.

La Banca et al. looked at instructional therapeutic play (ITP) as a method to help children better understand and master insulin therapy (Table 1). They found that ITP was an effective method to identify educational needs and associated care in school-aged children.

**Table 1:** Play therapy

**Study**	**Objective**	**Type**	**Key findings**	**Age range**
Henkemans et al. [17]	To assess the impact of a personal robot in providing diabetes self-management education to children with diabetes	RCT (*n* = 27)	Increased knowledge of diabetes self-management in the active group that was not found in the control group (*p* = 0.001)	7–12 years
Pélicand et al. [7]	To study the impact of recreational method and tools to assist education of children with diabetes on self-management	Qualitative study (*n* = 14)	Results showed that recreational teaching methods were effective in enabling the children to develop various treatment-related skills	10–12 years
Pennafort et al. [18]	(To) study the impact of toy use in education of children with diabetes on self-management	Qualitative study (*n* = 26)	The instructional toys were found to increase interest in the educational program, and improved their familiarity with self-management techniques	7–11 years
La Banca et al. [19]	To understand the experience of children with diabetes through instructional therapeutic play (ITP)	Qualitative Study (*n* = 8)	They found that children responded well to ITP, and it effectively allowed them to ease tensions and feel more in control of their disease	6–10 years

### Art therapy (3 papers)

Vanelli et al. studied the use of drawing to help develop the understanding of diabetes and its treatments in young diabetes patients. They found that children who were better able to draw “The human body as it is inside” would have a stronger understanding of their type 1 diabetes and the associated treatments.

Macdonald et al. studied the use of art therapy to help support young adults transitioning from paediatric services to adult diabetes care (Table 2). They looked to measure diabetes-related emotional distress and determined that art therapy was helpful in reducing that stress in 57% of the participants through a participant satisfaction evaluation form. Although their results were promising, they were limited by inconsistent attendance of sessions and a small sample size.

**Table 2:** Art therapy

**Study**	**Objective**	**Type**	**Key findings**	**Age range**
Vanellli et al. [20]	To study the effectiveness of drawing to test knowledge of diabetes in children with diabetes	Qualitative study (*n* = 68)	Drawing was an effective method to test children on their understanding of diabetes and related self-management techniques	5–14 years
Macdonald et al. [21]	To study the use of art therapy to assist in the transition of youth from paediatric to adult diabetic care	Qualitative study (*n* = 12)	Art therapy was helpful in reducing emotional stress	15–25 years
Harel et al. [22]	To evaluate the effect of art therapy in youth with poorly controlled type 1 diabetes	Retrospective report (*n* = 29)	Art therapy was found to improve disease management through improved compliance	6–14 years

Harel et al. studied the impact of intensive art therapy in youth with poorly controlled type 1 diabetes. They used art therapy as an intervention to help assist with behavioural difficulties, including needle phobia and lack of compliance with nutritional requirements. They found that improvement in disease management was achieved in 56% of the case group compared to 23% of the control group (29).

### Music therapy (1 papers)

Gelernter et al. studied the use of auditory guided imagery (AGI) on glucose levels, glycated haemoglobin and quality of life in children with diabetes. Their study compared the effects of AGI with music vs. background music only (Table 3). The study consisted of 13 patients between the ages of 7 and 16. They found that in both cases mean interstitial glucose concentration decreased, and HbA1c decreased in both groups, but more significantly in the AGI group (*p* = 0.04).

**Table 3:** Music therapy

**Study**	**Objective**	**Type**	**Key findings**	**Age range**
Gelernter et al. [23]	To study the effect of auditory guided imagery (AGI) in addition to a standard diabetes care program for youth	RCT (*n* = 13)	Listening to AGI has potential in improving glycaemic control and glucose levels in youth with T1DM	7–16 years

### Exercise therapy (3 papers)

Cafazzo et al. examined the use of a mobile health (mHealth) app to promote improved treatment adherence in young diabetes patients. Their app promoted rewards based on strong diagnostic readings, and they found that this incentivized improved behaviour and treatment adherence from the participants.

Lee et al. examined the impact of different types of exercise on insulin sensitivity and reducing total adiposity and ectopic fat in adolescents (Table 4). They compared aerobic and resistance exercise against each exercise type individually through a 6-month exercise routine (3 days a week, 180 min). Eighty-five participants completed the study, with 90% exercise attendance. They found that the combined exercise program was similarly beneficial compared to the separate programs for improving insulin sensitivity and reducing ectopic fat.

**Table 4:** Exercise therapy

**Study**	**Objective**	**Type**	**Key findings**	**Age range**
Cafazzo et al. [24]	To design, develop and pilot a mobile health app for the management of diabetes in adolescents	Pilot study (*n* = 20)	The app showed improvement in number of blood glucose measurements taken and improved satisfaction from participants	12–16 years
Lee et al. [25]	To compare the effectiveness of combined aerobic and resistance exercise to individual exercise of either type in treating youth diabetes	RCT (*n* = 118)	Combined exercise was similarly beneficial to aerobic or resistance exercise, with all three cases improving insulin sensitivity and reducing ectopic fat	12–17 years
Seeger et al. [26]	To evaluate the effect of an exercise training program in children with T1DM	Pilot study (*n* = 7)	Body characteristics and vascular health all showed positive improvement after the 18-week exercise program	8–12 years

Seeger et al. studied the impact of exercise on physical fitness and vascular function in children with diabetes. They used an 18-week program, and examined maximal oxygen consumption, brachial artery endothelial function, common carotid artery diameter, wall thickness and wall-to-lumen ratio. After the program, they found that maximal oxygen consumption improved (*p* = 0.039) and brachial artery endothelial function also improved (*p* = 0.038). Carotid artery diameter, wall thickness and wall-to-lumen ratio did not change with any significance (*p* = 0.26, 0.53 and 0.27, respectively).

## Discussion

In children with T1D, the literature supports the role of play therapy. Play therapy was used for education of patients with diabetes and showed that the patients tended to have a greater enjoyment of education. Children were more engaged in the play activities compared to the control; however, engagement did tend to fall over time. Pennafort et al. explored in more depth the effects of play therapy on children with DM by looking at children interacting with dolls and diabetic equipment. They found that doing so allows children to eliminate their doubts, explore their curiosities and alleviate fears associated with diabetes-related treatments.

Several studies identified positive impacts of art therapy in children diagnosed with T1D. The positive effects of art therapy include the reduction of stress in children, the provision of education and improving overall well-being. In high risk youth with diabetes and impaired glycaemic control, Harel et al. identified that art therapy can assist youth in expressing emotions without having to do it in words. By allowing children to use their imagination around various diabetes equipment they were able to create a new perspective and, therefore, increase adherence.

There was no direct research on music therapy in the paediatric population with diabetes. Gelerneter et al. showed that glycaemic control improved in their study. This study could be used to help pave the path further for research on the impact of music therapy. Therapies such as art, music and play therapy are process orientated in nature, and the benefits can be difficult to measure by quantitative means. With advancing technology and increased abilities to measure throughout the therapy process, these challenges may potentially be reduced or eliminated, allowing these therapies to be better measured against conventional diabetes treatment strategies.

This study was meant to focus exclusively on children with only diabetes and controlling for play, exercise, art and music therapy. A limitation of this study is that children with diabetes can be included in a multitude of other studies alongside children with other chronic diseases thus potentially broadening the impact that different therapy modalities can have.

## Conclusion

This paper summarizes the current literature of four different therapy modalities in children with T1D. At present, the research is most limited in regard to music therapy with the focus being on chronic diseases in adult population. A need for randomized controlled trials exists for looking at the four various therapies the effects of them on children with T1D. While various studies have been done and showed a positive effect on stress, understanding and engagement using play, art, music and exercise therapy, more needs to be done to look at the quantitative impact of them. Future directions of research could look at the impact of therapies on quantitative metrics of glycaemic control in children, such as the impact of play therapy on HbA1c or ‘time in range’ in participants given the increasing prevalence and availability of continuous glucose monitoring.
